# Bilateral Atypical Femoral Fractures in a Patient with Multiple Myeloma Treated with Intravenous Bisphosphonate Therapy

**DOI:** 10.1155/2014/452418

**Published:** 2014-07-22

**Authors:** Ichiro Tonogai, Tomohiro Goto, Daisuke Hamada, Toshiyuki Iwame, Shinji Yoshioka, Takahiko Tsutsui, Yuichiro Goda, Hiroshi Egawa, Koichi Sairyo

**Affiliations:** Department of Orthopedics, Institute of Health Biosciences, The University of Tokushima Graduate School, 3-18-15 Kuramoto, Tokushima 770-8503, Japan

## Abstract

Bisphosphonates are currently the standard approach to managing bone disease in multiple myeloma. Bisphosphonates have high bone affinity that inhibits osteoclastic activity and additionally reduces the growth factors released from malignant or osteoblastic cells, thereby impairing abnormal bone remodeling which leads to osteolysis. However, patients of multiple myeloma may be at a higher risk of atypical femoral fractures because the treatment for malignant myeloma requires notably higher cumulative doses of bisphosphonates. Here we present a patient with bilateral atypical femoral fractures and multiple myeloma treated with intravenous bisphosphonate therapy.

## 1. Introduction

Multiple myeloma accounts for around 1% of all types of malignancy and slightly more than 10% of hematologic malignancies [1]. Bone lesions occur in virtually all patients with advanced-stage multiple myeloma, which amounts to almost 60,000 people globally each year [2]. The aggressive features of multiple myeloma bone lesions also contribute significantly to its poor prognosis [3–5].

Bisphosphonates are now commonly used in the treatment of multiple myeloma and have been shown to be highly effective for reducing the risk of skeletal-related events by breaking the cycle of bone resorption. However, recent studies have raised concerns that long-term bisphosphonate therapy may cause atypical femoral fractures potentially due to impaired bone remodeling [6, 7]. Atypical femoral fractures in multiple myeloma patients treated with bisphosphonates are relatively rare because of the small number of affected patients. In this report, we describe a rare case of bilateral atypical femoral fractures caused by long-term intravenous bisphosphonate treatment for multiple myeloma.

## 2. Case Report

**Figure 1:**
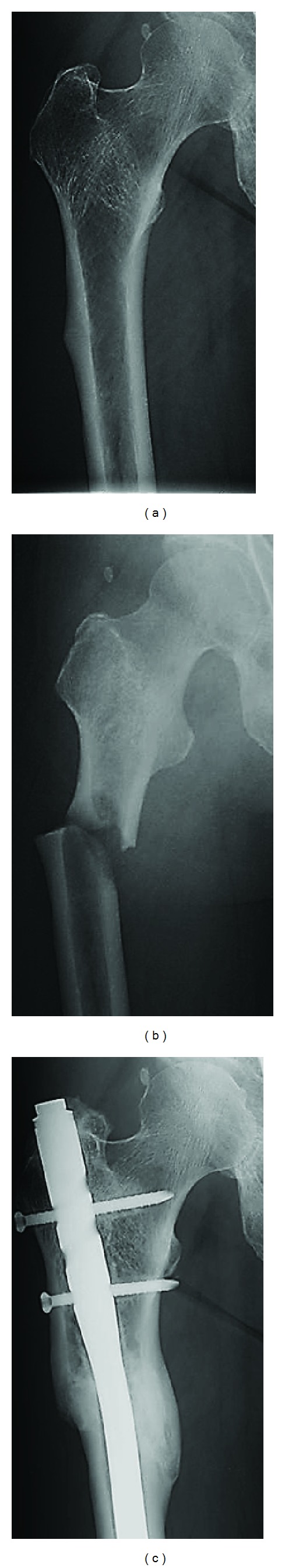
Radiographs of the right femur before and after the atypical fracture. (a) Radiograph at the initial visit to a local hospital revealed diffuse cortical thickening and beaking of the lateral cortex in the subtrochanteric region of the right femur. (b) A short oblique fracture with a medial spike is visible in the subtrochanteric region of the right femur. (c) The fracture was stabilized by intramedullary nailing, and union is evident within 4 weeks of the right femoral fracture.

**Figure 2:**
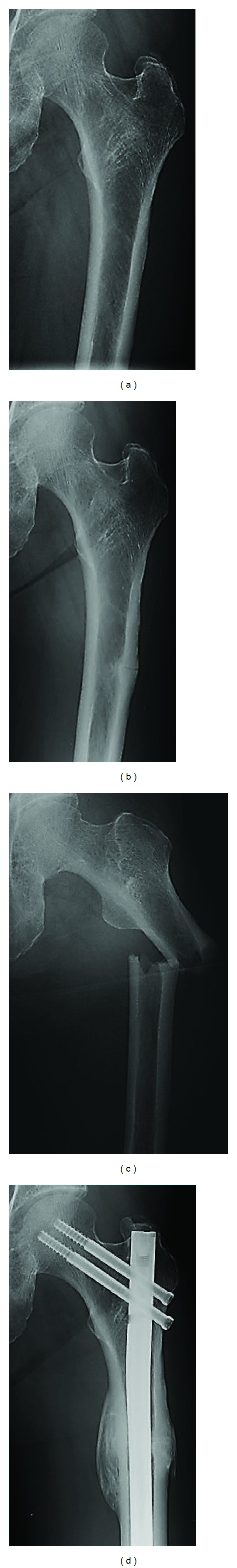
Radiographs of the left femur before and after the atypical fracture. (a) No abnormal findings are evident at the time of right femoral atypical fracture. (b) Radiograph at initial presentation to us showed diffuse cortical thickening and beaking of the lateral cortex in the subtrochanteric region of the left femur. (c) A left subtrochanteric femoral fracture with a unicortical beak, hypertrophied cortices, and a transverse pattern is evident. (d) The fracture was fixed by intramedullary nailing and union is evident within 4 weeks of the left femoral fracture.

Our female patient was first diagnosed with multiple myeloma at the age of 39 years. She had been receiving intravenous bisphosphonates injection (incadronate 10 mg/month × 70 doses and zoledronate 4 mg/3 months × 20 doses) for around 11 years (from ages 44 to 56) to treat multiple myeloma. At age 53, she presented with prodromal right thigh pain and was referred to the orthopaedic department of a local hospital, but a precise diagnosis was not made. At this time, she had received 70 doses of incadronate for approximately 6 years and 8 doses of zoledronate for approximately 2 years. Radiographs revealed lateral cortical thickening in the right subtrochanteric femoral shaft from a retrospective viewpoint (Figure 1(a)). Three months later, she sustained a right atraumatic subtrochanteric femoral shaft fracture. This atypical fracture was a short oblique fracture with a medial spike, occurring below the lesser trochanter with evidence of focal hypertrophy of the lateral cortex (Figure 1(b)). Radiographs of the left femur revealed no abnormal findings (Figure 2(a)). The right femoral fracture was treated by intramedullary nailing, and bone union was obtained 4 months postoperatively (Figure 1(c)).

Two and a half years after the right femoral fracture (at age 56), she experienced antecedent pain in the left thigh and was referred to our hospital. By that time she had received 70 doses of incadronate for approximately 6 years and 20 doses of zoledronate for approximately 5 years, and again radiographs showed lateral cortical thickening in the left subtrochanteric femoral shaft (Figure 2(b)). Her previous history, previous X-rays, and bone mineral density data suggested no evidence of bone abnormalities associated with osteoporosis. We assumed that another atypical femoral fracture of the left femur had occurred because of the antecedent thigh pain and typical radiographic findings. We recommended she discontinues intravenous bisphosphonates treatment. Three months later, she sustained a left subtrochanteric femoral shaft fracture after a low-energy fall from standing or lower height. This latest atypical fracture was a transverse fracture, occurring below the lesser trochanter with evidence of focal hypertrophy of the lateral cortex (Figure 2(c)). She underwent intramedullary nailing of the left femur, and bone union was evident within about 4 months (Figure 2(d)).

Her postoperative course was uneventful after this final surgery. At last follow-up, about 2 years after the surgery, she could walk without a walking stick and independently perform activities of daily living.

## 3. Discussion

Multiple myeloma is a cancer characterized by uncontrolled proliferation of clonal plasma cells. Myeloma plasma cells exist in the bone marrow microenvironment and secrete factors that stimulate osteoclast-mediated osteolysis and suppress osteoblastic differentiation, leading to devastating bone destruction and rapid bone loss [1, 8–10]. Bisphosphonates are currently the standard treatment for managing bone disease in multiple myeloma. They function by mainly impairing malignant osteolysis through suppressing osteoclast activity, inducing apoptosis of osteoclasts, and impairing multiple myeloma growth that can result in skeletal-related events such as fracture and bone pain [11]. Bisphosphonates, especially nitrogen-containing ones like zoledronate, may have inherent anticancer actives [11, 12].

On the other hand, cumulative doses of bisphosphonates for malignant myeloma treatment tend to be notably higher than those for osteoporosis treatment. Long-term bisphosphonates therapy severely suppresses bone turnover through inhibiting osteoclasts and results in the failure to repair microcracks in the cortical bone of animal models, which may lead to the accumulation of damage and reduced cortical bone toughness [13, 14]. It is thought that impairment of such bone remodeling by long-term cumulative bisphosphonates treatment leads to atypical fractures.

We have presented a rare case of bilateral atypical fractures in a patient with multiple myeloma treated with intravenous bisphosphonates therapy. To our knowledge, only 4 cases in 3 previous reports of atypical femoral fractures caused by long-term bisphosphonate therapy for multiple myeloma have been reported (Table 1) [15–17]. In these reports including the present case, pamidronate, incadronate, and/or zoledronate were used for multiple myeloma treatment. The potency of these 3 agents in bone resorption is 10–100, 100–1,000, and >10,000 times stronger than that of etidronate, respectively [18]. The mean (±SD) duration of bisphosphonates treatment in these reports including the present case was 105.6 ± 14.4 months and that of cumulative doses of bisphosphonates was 78.5 ± 12.7 months.

The current guidelines from the Society of Clinical Oncology for intravenous bisphosphonate administration in patients with multiple myeloma recommend 90 mg pamidronate or 4 mg zoledronic acid every 3-4 weeks and considering discontinuation of intravenous bisphosphonates after 2 years if a responsive disease state or stable disease state is evident [19]. In the present case, it was important that we were aware of the possibility of atypical fracture and that bisphosphonates should be discontinued at the initial presentation of prodromal left thigh pain and at the time of right atypical femoral fracture. Advanced imaging such as bone scanning or MRI should be considered in similar cases. When considering that around 50% of such atypical fractures occur bilaterally [20], prophylactic intramedullary nailing might be prudent. Atypical femoral fractures, together with other possible complications of bisphosphonates use, must be balanced against the obvious benefits of reducing skeletal events in multiple myeloma patients.

## Figures and Tables

**Table 1:** Summary of case reports on atypical fractures in patients with multiple myeloma treated with bisphosphonates therapy.

Author and year	Age (yr)	Sex	Fracture side	Duration of treatment with bisphosphonates (mo)	Number of bisphosphonate doses
Grasko et al., 2009 [15]	66	M	L	96	88 (47 pamidronate, 41 zoledronate)

Puhaindran et al., 2011 [16]	64	F	R	103	58 (all pamidronate)

Chang et al., 2012 [17]	N/A	N/A	Bilateral	N/A	N/A (pamidronate and/or zoledronate)
	N/A	N/A	Bilateral	N/A	N/A (pamidronate and/or zoledronate)

Present case (same patient)	53	F	R	94	78 (70 incadronate, 8 zoledronate)
	56	F	L	130	90 (70 incadronate, 20 zoledronate)

M: male; F: female; R: right; L: left; N/A: not available.
